# Chemical Synthesis and Oxide Dispersion Properties of Strengthened Tungsten via Spark Plasma Sintering

**DOI:** 10.3390/ma9110879

**Published:** 2016-10-28

**Authors:** Xiao-Yu Ding, Lai-Ma Luo, Hong-Yu Chen, Xiao-Yong Zhu, Xiang Zan, Ji-Gui Cheng, Yu-Cheng Wu

**Affiliations:** 1 School of Materials Science and Engineering, Hefei University of Technology, Hefei 230009, China; dingxiaoyu0903@126.com (X.-Y.D.); 15222918166@163.com (H.-Y.C.); zhuxiaoyong@hfut.edu.cn (X.-Y.Z.); zanx@hfut.edu.cn (X.Z.); jgcheng63@sina.com (J.-G.C.); 2 Laboratories of Nonferrous Metal Material and Processing Engineering of Anhui Province, Hefei 230009, China

**Keywords:** oxide dispersion-strengthened W, wet chemical method, spark plasma sintering, irradiation behavior

## Abstract

Highly uniform oxide dispersion-strengthened materials W–1 wt % Nd_2_O_3_ and W–1 wt % CeO_2_ were successfully fabricated via a novel wet chemical method followed by hydrogen reduction. The powders were consolidated by spark plasma sintering at 1700 °C to suppress grain growth. The samples were characterized by performing field emission scanning electron microscopy and transmission electron microscopy analyses, Vickers microhardness measurements, thermal conductivity, and tensile testing. The oxide particles were dispersed at the tungsten grain boundaries and within the grains. The thermal conductivity of the samples at room temperature exceeded 140 W/m·K. The tensile tests indicated that W–1 wt % CeO_2_ exhibited a ductile–brittle transition temperature between 500 °C and 550 °C, which was a lower range than that for W–1 wt % Nd_2_O_3_. Surface topography and Vickers microhardness analyses were conducted before and after irradiations with 50 eV He ions at a fluence of 1 × 10^22^ m^−2^ for 1 h in the large-powder material irradiation experiment system. The grain boundaries of the irradiated area became more evident than that of the unirradiated area for both samples. Irradiation hardening was recognized for the W–1 wt % Nd_2_O_3_ and W–1 wt % CeO_2_ samples.

## 1. Introduction

The development of fusion technology for electrical power production is one of the major challenges faced in the 21st century [1]. Finding the ideal plasma facing material is one of the most crucial issues for fusion power plants [2]. In the International Thermonuclear Experimental Reactor, the divertor material in the deuterium discharge phase is purely tungsten because of this material’s low tritium retention [3]. However, problems such as coarse grain, inherent high ductile–brittle transition temperature (DBTT), poor ductility, low fracture toughness, recrystallization brittleness, and radiation-induced brittleness inhibit the material from meeting the harsh wall loading requirements of future fusion reactors [4,5]. Although W-based alloys retain structural integrity over their lifetime, they are generally not utilized for structural purposes because of their brittleness at low temperatures [6]. Therefore, novel tungsten materials with improved ductility and stability against high temperatures and neutron radiation must be developed. Second-phase particle dispersoids are effective in mitigating the aforementioned problems. In general, Y_2_O_3_, La_2_O_3_, ZrC, TiC, and other similar dispersoids are commonly used because they can inhibit grain growth during sintering, hinder grain boundary sliding, and stabilize the microstructure. These well-dispersed nanomaterials can also act as points of annihilation for radiation-induced defects [7,8]. Rare-earth oxide particles are particularly effective because they can gather solutes produced by strong rare-earth–oxygen interactions [9]. These oxide dispersion-strengthened (ODS) composite materials are typically prepared by mechanical alloying. However, the milling process results in a detrimental phase because of the wearing of the milling equipment and the media. A wet chemical process can effectively fabricate and prepare complex nanomaterials while maintaining a precise composition with high purity and homogeneity [10,11,12]. A major challenge in the sintering of nanopowders is the achievement of full densification while preserving the nanoscale. Most tungsten-based alloys are fabricated by powder metallurgy methods because of its high melting point of 3410 °C. An example of recent technological innovation is the use of spark plasma sintering (SPS), which allows for the consolidation of powder materials into dense fine-grained products at lower sintering temperatures [13,14,15].

Compared with other oxide particles, Nd_2_O_3_ and CeO_2_ have higher melting points and thermal conductivities and are therefore promising candidates for plasma facing materials. In this study, highly uniform dense fine-grained ODS tungsten (ODS-W) samples were fabricated by performing the wet chemical method and the SPS technique on the reactions between tungsten, neodymium, and cerium salts in an aqueous solution at room temperature (RT). The two components were mixed at the molecular level, resulting in a highly homogenous precursor. The effects of adding Nd_2_O_3_ and CeO_2_ particles on the consolidation behavior, microstructure, hardness, tensile behavior, and thermal conductivity of ODS tungsten samples were comparatively investigated.

## 2. Experimental Procedure

Ammonium paratungstate hydrate (APT, Aladdin, Los Angeles, CA, USA) was suspended in an aqueous solution of neodymium nitrate hexahydrate (cerium nitrate hexahydrate) to synthesize neodymium (cerium)-doped tungsten precursors with neodymium (cerium) content corresponding to W–1 wt % Nd_2_O_3_ and W–1 wt % CeO_2_, respectively. Nitric acid (65%) was added under vigorous stirring; then, ethanol was added, to double the total solvent mass. The suspension was stirred for another 3 h to slow down the condensation of tungstic acid and limit particle growth. The powder was calcined under nitrogen atmosphere at 450 °C for 1 h, wherein the powder was transformed into an oxide mixture. Then, the precursor was reduced by high-purity hydrogen in a single-tube electrically heated furnace (GSL-1400, Hefei Ke Jing Materials technology Co. Ltd., Hefei, China). The boat containing the precursor was placed in the central section of the furnace tube and heated at 800 °C at a heating rate of 5 °C/min in a gas flow. The mixture was maintained at the aforementioned temperature for 6 h. Afterwards, the sample, which was under a hydrogen flow, was cooled to RT. The as-received ammonium paratungstate was reduced to pure tungsten powder under the same conditions in a separate experiment.

**Figure 1:**
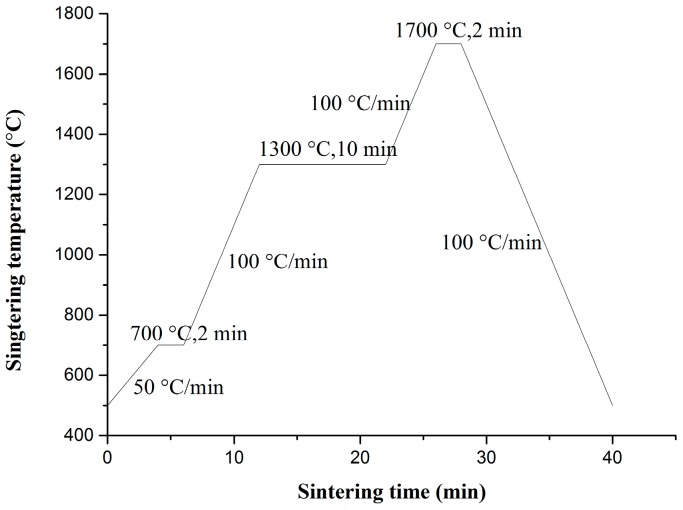
Temperature profile of the spark plasma sintering (SPS) process.

The reduced powders were first pressed under air in a graphite die with a 20 mm inner diameter and were subsequently sintered under vacuum using SPS (LABOX-350, Sinter Land Inc., Nagaoka City, Japan). The temperature profile of the sintering program in this study is illustrated in Figure 1. Density measurements of sintered samples were performed using the Archimedes method. The theoretical density of the ODS-W composites was calculated from the fractional and theoretical densities of each component. Polished sintered samples were subjected to Vickers microhardness testing (MH-3L, Shanghai Everone Precision Instruments Co. Ltd., Shanghai, China) at 200 g loads and a dwell time of 20 s at RT. Tensile testing was performed using the Instron testing machine (Instron 5967, Instron, Norwood, MA, USA). The tensile test specimens were dog-bone-shaped with an overall length of 16 mm, a gauge length of 5 mm, and an effective cross section of 4 mm × 0.75 mm. The tensile sample was placed on a pull rod and was heated at various temperatures from 500 °C to 700 °C in a resistance heating furnace. Thermal conductivity test was performed using the laser flash thermal analyzer (LFA 457, Netzsch, Selb, Germany). Measurements of the thermal diffusivity of the materials were conducted using the laser flash technique with disk samples of 6 mm diameter and 2–3 mm thickness. Microstructures and particle morphologies of the precursor and reduced powders were investigated by field emission scanning electron microscopy (FE-SEM, SU8020; Hitachi, Tokyo, Japan) and transmission electron microscopy (TEM, JEF-2100F, JEOL, Tokyo, Japan). X-ray diffraction (D/MAX 2500 V, Rigaku, Tokyo, Japan) was employed to characterize the prepared materials. The morphologies of the SPS-sintered bulk tungsten materials were characterized by field emission scanning electron microscopy (FE-SEM) and transmission electron microscopy (JEF-2100F, JEOL). Surface topography and Vickers microhardness examinations were conducted before and after irradiations with 50 eV He ions at a fluence of 1 × 10^22^ m^−2^ for 1 h in the large-powder material irradiation experiment system.

## 3. Results and Discussion

**Figure 2:**
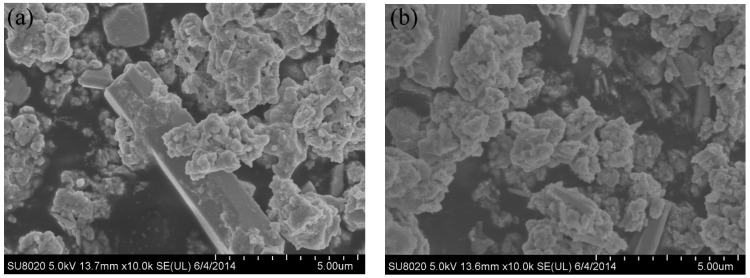
Scanning electron microscopy (SEM) micrographs of precursor powders before reduction in hydrogen. (**a**) W–1 wt % Nd_2_O_3_; (**b**) W–1 wt % CeO_2_.

**Figure 3:**
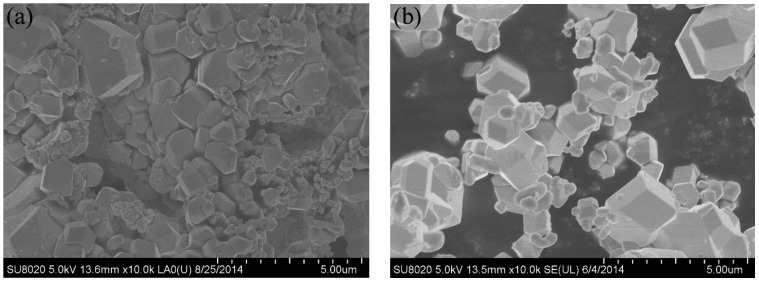
SEM images of the reduced powders showing bimodal morphology. (**a**) W–1 wt % Nd_2_O_3_; (**b**) W–1 wt % CeO_2_.

**Figure 4:**
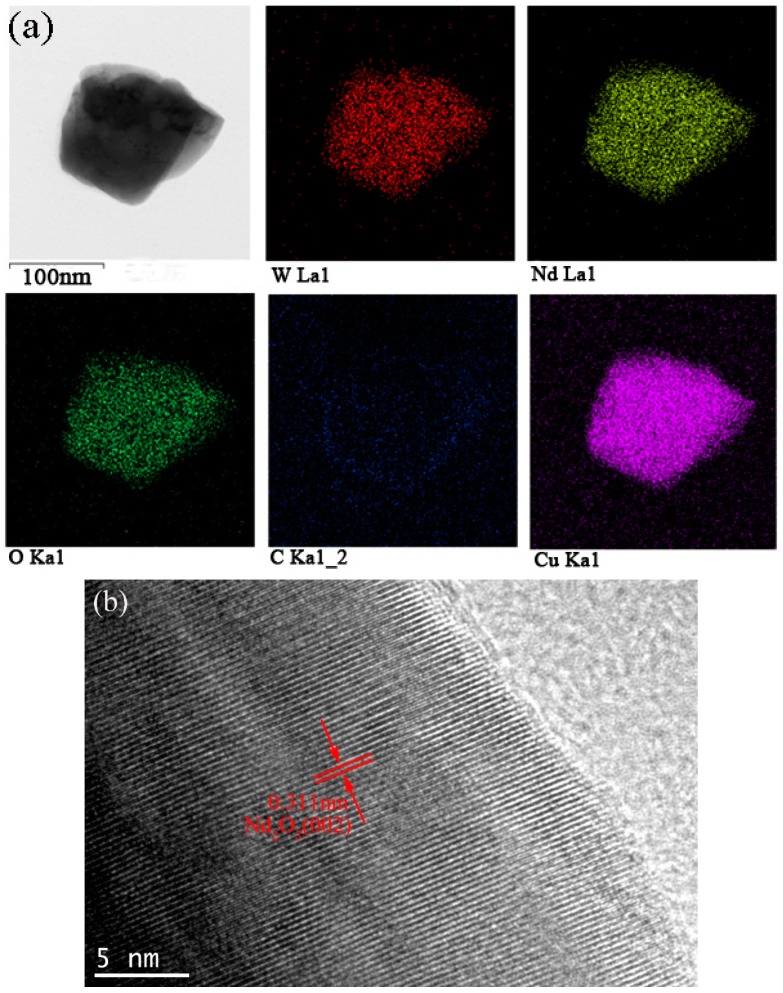
Transmission electron microscopy (TEM) images of the as-synthesized W–1 wt % Nd_2_O_3_ powders. (**a**) Energy dispersive X-ray spectroscopy (EDS) mapping images showing elemental distributions; (**b**) High-resolution transmission electron microscopy (HR-TEM) image showing the interplanar crystal spacing.

The synthesized powder precursors were analyzed via FE-SEM. As shown in Figure 2, the precursor powders are agglomerated together and consist of agglomerates with a size distribution of 5–10 µm, which were much smaller compared with a chemically synthesized APT-La precursor reported to have a particle size distribution up to 70 µm [16,17]. Reduction of the precursor in nitrogen followed by hydrogen resulted in the formation of a powder consisting of particles with two different morphologies, namely, (i) large particles with a polygonal morphology; and (ii) fine particles with a cubic shape, as shown in Figure 3. The bimodal size distribution exhibited a significant contribution to the high packing density of the powders. Qualitatively, neodymium, and oxygen were confirmed in these particles via energy dispersive X-ray spectroscopy (EDS) analysis using transmission electron microscopy. As shown in Figure 4a, the mapping demonstrated the formation of a uniform mixture of Nd^3+^ and tungsten ions. Cu detected was the copper grid. The HR-TEM images demonstrated the interplanar crystal spacing and clarified the phase of Nd_2_O_3_ particles as shown in Figure 4b. Reduced powder from W–1 wt % CeO_2_ also exhibited the same homogeneous distribution of elements.

**Figure 5:**
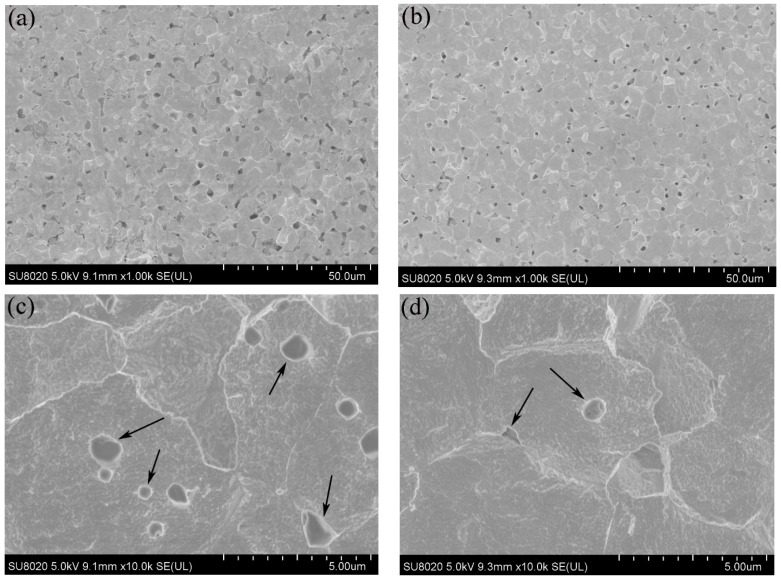
SEM micrographs of the polished and etched surfaces. (**a**,**c**) W–1 wt % Nd_2_O_3_; (**b**,**d**) W–1 wt % CeO_2_.

**Figure 6:**
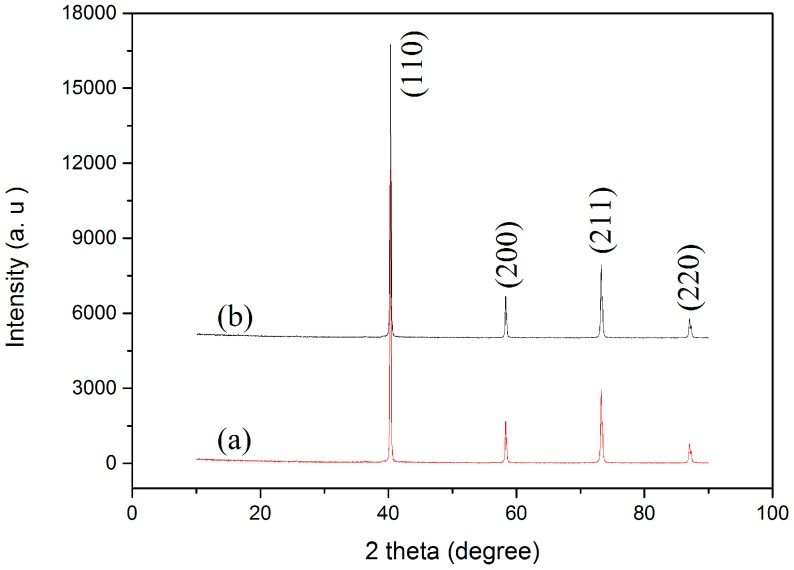
X-ray powder diffraction (XRD) patterns of (**a**) W–1 wt % Nd_2_O_3_; (**b**) W–1 wt % CeO_2_ composites sintered by SPS.

Figure 5 shows the SEM images of the polished and etched surfaces of the SPS-sintered samples. The low-magnification SEM images (Figure 5a,b) showed oxide distribution in the sintered samples. This phenomenon revealed that the surface of the SPS-sintered samples was nearly fully dense. The relative density of the W–1 wt % Nd_2_O_3_ sample was approximately 96.5%. However, the relative density of the W–1 wt % CeO_2_ sample sintered at the same condition was only approximately 95.9%. This result indicated that the W–1 wt % Nd_2_O_3_ sample exhibited better sinterability compared with the W–1 wt % CeO_2_ sample. The thermal conductivity of W–1 wt % CeO_2_ was lower than that of W–1 wt % Nd_2_O_3_ at RT. This result may have originated from the low relative density. The high-magnification SEM images (Figure 5c,d) showed that the oxide particles were dispersed at the tungsten grain boundaries, as well as within the grains (marked by arrows). The highly uniform distribution of oxides in the sintered bulk material is an indication of the homogeneous mixing of W and Nd (Ce) during precursor synthesis. X-ray powder diffraction (XRD) analysis (Figure 6) showed that no major phase change occurred for both samples during the SPS process. It showed the advantage of the wet chemical process compared with milling procedures, for avoiding formation of WC impurities from the jar and the balls [18]. The same phenomenon was observed during the mechanical alloying of other materials [19].

**Figure 7:**
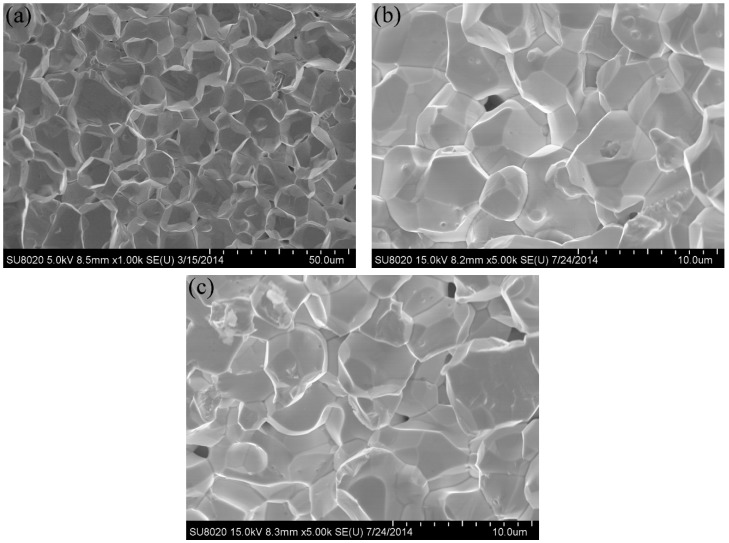
SEM micrographs of the fracture surfaces after tensile test at room temperature for: (**a**) pure W; (**b**) W–1 wt % Nd_2_O_3_; (**c**) W–1 wt % CeO_2_.

The fracture surface of the W–1 wt % Nd_2_O_3_ and W–1 wt % CeO_2_ samples is presented in Figure 7b,c. The result of pure tungsten is also shown for comparison (Figure 7a). The fresh fracture surface was obtained by breaking the specimen after the tensile test at RT. Figure 7a shows an average pure tungsten grain size of 10 µm. The average grain size of sintered W–1 wt % Nd_2_O_3_ and W–1 wt % CeO_2_ was approximately 4 µm (Figure 7b,c). This value was smaller than that of pure tungsten, as indicated by data listed in Table 1. These results demonstrated that oxide particles were effective in hindering the grain growth of tungsten during the sintering process. The fracture surface was a typical intergranular failure. This finding indicates that these samples are brittle at RT.

**Figure 8:**
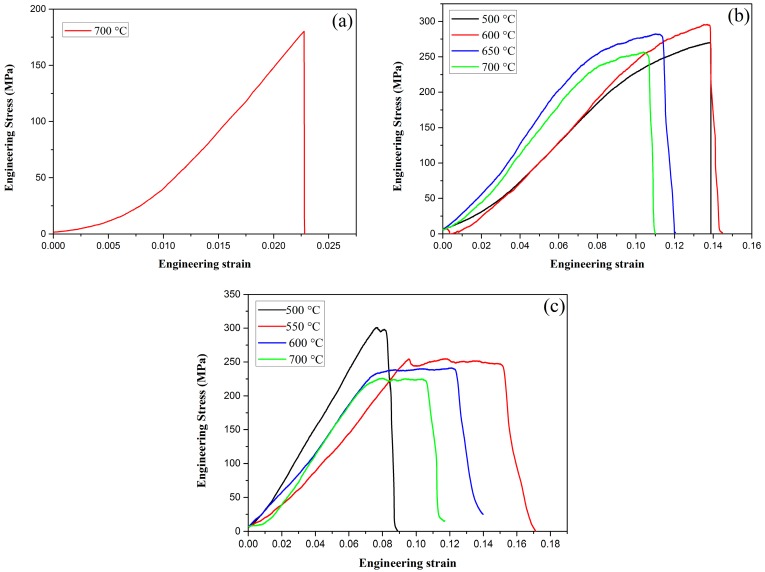
Tensile behaviors for (**a**) pure W; (**b**) W–1 wt % Nd_2_O_3_; (**c**) W–1 wt % CeO_2_.

Tensile testing was performed using the Instron testing machine at RT at a loading rate of 0.05 mm/min. The engineering stress–strain curves of pure W, W–1 wt % Nd_2_O_3_, and W–1 wt % CeO_2_ at various temperatures are presented in Figure 8. As shown in Figure 8a, pure W showed a typical brittle fracture at 700 °C. The fracture strength is only approximately 185 MPa. However, ductility can be drastically improved by the addition of oxide particles, such as Nd_2_O_3_ or CeO_2_, as shown in Figure 8b,c. Obviously, the tensile strength of ODS-W was higher than that of pure W. This result indicates that dispersing particles in the grain interior and at the grain boundaries is an effective approach to increase the tensile strength. The deterioration of tensile properties at high temperatures must be caused by the softening and embrittlement of the matrix phase by oxidation during the test conducted in air. Liu et al. adopted micro-alloying by adding a small amount of Zirconium (Zr) to further improve the ultimate tensile strength of ODS tungsten, which was a successful attempt to obtain high performance W materials [20].

**Figure 9:**
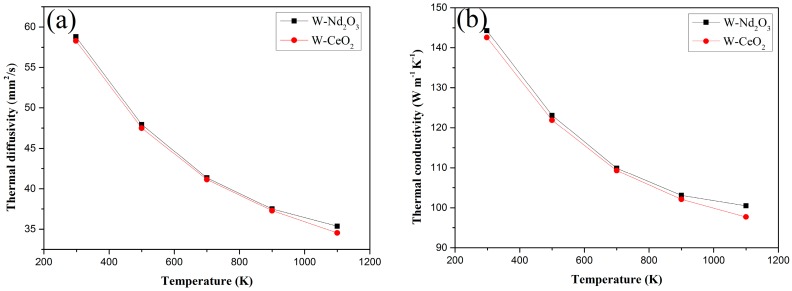
Thermal diffusivities and thermal conductivities versus temperature of W–1 wt % Nd_2_O_3_ and W–1 wt % CeO_2_ composites. (**a**) Thermal diffusivities; (**b**) thermal conductivities.

The thermal diffusivities and thermal conductivities of the Nd_2_O_3_/W and CeO_2_/W composites from RT to 1100 K are shown in Figure 9a,b respectively. The thermal diffusivity of Nd_2_O_3_/W drastically decreased from RT to 1100 K, whereas the CeO_2_/W composites decreased with the same trend as the temperature increased in the testing temperature range. The thermal conductivity of both samples decreased with increasing temperatures. However, the thermal conductivity of both samples is above 140 W/m·K at RT. This value is higher than that of W–1 wt % La_2_O_3_ and W–1 wt % Y_2_O_3_ samples in similar studies [21]. 

**Figure 10:**
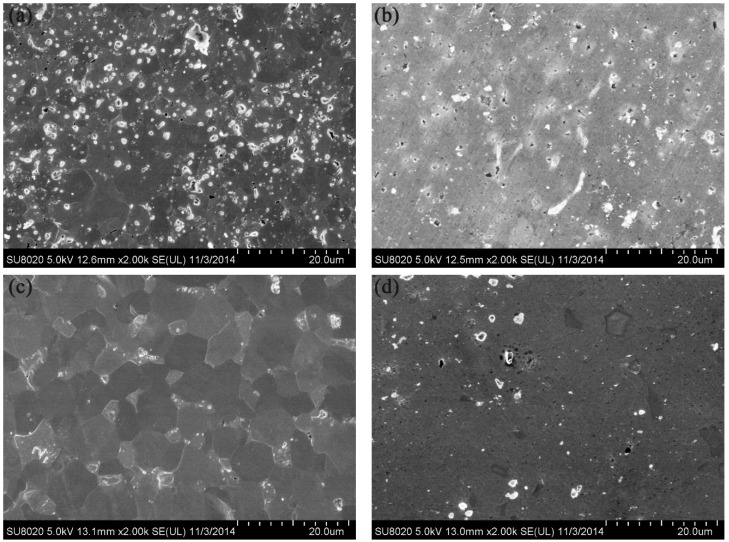
SEM images of samples exposed to 50 eV He-ions to the fluence of 1 × 10^22^ m^−2^ for 1 h. (**a**) Irradiated area of W–1 wt % Nd_2_O_3_; (**b**) Unirradiated area of W–1 wt % Nd_2_O_3_; (**c**) Irradiated area of W–1 wt % CeO_2_; (**d**) Irradiated area of W–1 wt % CeO_2_.

Helium atoms, compared with hydrogen atoms, cause more material damages and He-related defects, such as He bubbles and nanostructures (fuzz). This phenomenon results from He irradiation/exposure. He irradiation and exposure strongly influence surface properties. Figure 10 shows the SEM images of W–1 wt % Nd_2_O_3_ and W–1 wt % CeO_2_ samples exposed to 50 eV He ions at a fluence of 1 × 10^22^ m^−2^ for 1 h in the large-powder material irradiation experiment system. No significant surface damage was observed. However, the grain boundaries of the irradiated area became more evident compared with the unirradiated area for both samples. The Vickers microhardness numbers of the W–1 wt % Nd_2_O_3_ and W–1 wt % CeO_2_ samples before irradiation were 349 and 305, respectively. By contrast, the Vickers microhardness numbers of the W–1 wt % Nd_2_O_3_ and W–1 wt % CeO_2_ samples after irradiation were 374 and 340, respectively. The amount of radiation hardening, ∆HV, was 25 for W–1 wt % Nd_2_O_3_ and 35 for W–1 wt % CeO_2_. This result indicates that radiation hardening occurred in both samples. Hence, it may be stated that, even though a low dose of He irradiation on the present samples does not produce severe damage, it increases the hardness of the materials, which may be due to the irradiation-induced defects [22]. 

## 4. Conclusions

ODS materials W–1 wt % Nd_2_O_3_ and W–1 wt % CeO_2_ were successfully synthesized via a novel wet chemical method. These materials were sintered using the SPS technique in a shorter time compared with conventional sintering methods. This effect allowed the avoidance of substantial grain growth. The relative density of the W–1 wt % Nd_2_O_3_ sample was approximately 96.5%. This value is higher than that of W–1 wt % CeO_2_. This result indicated that the W–1 wt % Nd_2_O_3_ sample exhibited better sinterability than W–1 wt % CeO_2_. The dispersion of Nd_2_O_3_ and CeO_2_ particles can significantly inhibit grain growth of tungsten during consolidation. The tensile tests indicated that ductility can be drastically improved by adding oxide particles. W–1 wt % CeO_2_ exhibited a DBTT between 500 °C and 550 °C. This value is lower than that of W–1 wt % Nd_2_O_3_. The thermal conductivity of these samples was higher than 140 W/m·K at RT. Surface topography and Vickers microhardness examinations were conducted before and after irradiations with 50 eV He ions at a fluence of 1 × 10^22^ m^−2^ for 1 h in the large-powder material irradiation experiment system. The grain boundaries of the irradiated area became more evident compared with the unirradiated area for both samples. Irradiation hardening was recognized for W–1 wt % Nd_2_O_3_ and W–1 wt % CeO_2_ samples.

## Figures and Tables

**Table 1:** Density, grain size, and Vickers microhardness (H_V200g_) of pure W, W–1 wt % Nd_2_O_3_, and W–1 wt % CeO_2_ samples sintered by spark plasma sintering (SPS).

Materials	Density (g/cm^3^)	Relative Density (%)	Grain Size (μm)	Hardness (H_V200g_)
W	18.5	95.8	10	270
W–1 wt % Nd_2_O_3_	18.3	96.5	4	349
W–1 wt % CeO_2_	18.2	95.9	4	305

## References

[1] Terentyev D., Dubinko V., Bakaev A., Zayachuk Y., Renterghem W.V., Grigorev P. (2014). Dislocations mediate hydrogen retention in tungsten. Nucl. Fusion.

[2] Shu X.Y., Qiu H.X., Huang B., Gu Z.X., Yang J.J., Liao J.L., Yang Y.Y., Liu N., Tang J. (2013). Preparation and characterization of potassium doped tungsten. J. Nucl. Mater..

[3] Kajita S., Temmerman G.D., Morgan T., Eden S., Kruif T., Ohno N. (2014). Thermal response of nanostructured tungsten. Nucl. Fusion.

[4] Luo L.M., Tan X.Y., Chen H.Y., Luo G.N., Zhu X.Y., Cheng J.G., Wu Y.C. (2015). Preparation and characteristics of W–1 wt.% TiC alloy via a novel chemical method and spark plasma sintering. Powder Technol..

[5] Bolokang A.S., Phasha M.J., Maweja K., Bhero S. (2012). Structural characterization of mechanically milled and annealed tungsten powder. Powder Technol..

[6] Xie Z.M., Zhang T., Liu R., Fang Q.F., Miao S., Wang X.P., Liu C.S. (2015). Grain growth behavior and mechanical properties of zirconium micro-alloyed and nano-size zirconium carbide dispersion strengthened tungsten alloys. Int. J. Refract. Met. Hard Mater..

[7] Xia M., Yan Q.Z., Xu L., Guo H.Y., Zhu L.X., Ge C.C. (2013). Bulk tungsten with uniformly dispersed La_2_O_3_ nanoparticles sintered from co-precipitated La_2_O_3_/W nanoparticles. J. Nucl. Mater..

[8] Kurishita H., Matsuo S., Arakawa H., Kobayashi S., Nakai K., Takida T., Takebe K., Kawai M. (2008). Superplastic deformation in W–0.5 wt.% TiC with approximately 0.1 µm grain size. Mater. Sci. Eng. A.

[9] Liu G., Zhang G.J., Jiang F., Ding X.D., Sun Y.J., Sun J., Ma E. (2013). Nanostructured high-strength molybdenum alloys with unprecedented tensile ductility. Nat. Mater..

[10] Xu L., Yan Q.Z., Xia M., Zhu L.X. (2013). Preparation of La_2_O_3_ doped ultra-fine W powders by hydrothermal-hydrogen reduction process. Int. J. Refract. Met. Hard Mater..

[11] Wahlberg S., Yar M.A., Abuelnaga M.O., Salem H.G., Johnsson M., Muhammed M. (2012). Fabrication of nanostructured W–Y_2_O_3_ materials by chemical methods. J. Mater. Chem..

[12] Chaudhuri R.G., Paria S. (2012). Core/Shell Nanoparticles: Classes, Properties, Synthesis Mechanisms, Characterization, and Applications. Chem. Rev..

[13] Tshephe T.S., Olubambi P.A., Sigalas I., Ozoemena K.I., Garrett J., Sule R. (2015). Characterization of TiO_2_–MnO_2_ composite electrodes synthesized using spark plasma sintering technique. Powder Technol..

[14] Ding L., Xiang D.P., Li Y.Y., Li C., Li J.B. (2012). Effects of sintering temperature on fine-grained tungsten heavy alloy produced by high–energy ball milling assisted spark plasma sintering. Int. J. Refract. Met. Hard Mater..

[15] Xie Z.M., Liu R., Fang Q.F., Zhou Y., Wang X.P., Liu C.S. (2014). Spark plasma sintering and mechanical properties of zirconium micro-alloyed tungsten. J. Nucl. Mater..

[16] Yar M.A., Wahlberg S., Bergqvist H., Salem H.G., Johnsson M., Muhammed M. (2011). Spark plasma sintering of tungsten–yttrium oxide composites from chemically synthesized nanopowders and microstructural characterization. J. Nucl. Mater..

[17] Yar M.A., Wahlberg S., Bergqvist H., Salem H.G., Johnsson M., Muhammed M. (2011). Chemically produced nanostructured ODS–lanthanum oxide–tungstencomposites sintered by spark plasma. J. Nucl. Mater..

[18] Veleva L., Oksiuta Z., Vogt U., Baluc N. (2009). Sintering and characterization of W–Y and W–Y_2_O_3_ materials. Fusion Eng. Des..

[19] Kiran U.R., Kumar M.P., Sankaranarayana M., Singh A.K., Nandy T.K. (2015). High energy milling on tungsten powders. Int. J. Refract. Met. Hard Mater..

[20] Liu R., Xie Z.M., Hao T., Zhou Y., Wang X.P., Fang Q.F., Liu C.S. (2014). Fabricating high performance tungsten alloys through zirconium micro-alloying and nano-sized yttria dispersion strengthening. J. Nucl. Mater..

[21] Liu Y., Zhou Y., Hao T., Zhang T., Wang X.P., Liu C.S., Fang Q.F. (2012). Microwave synthesis and properties of fine–grained oxides dispersion strengthened tungsten. J. Nucl. Mater..

[22] Battabyal M., Spätig P., Murty B.S., Baluc N. (2014). Investigation of microstructure and microhardness of pure W and W–2Y_2_O_3_ materials before and after ion-irradiation. Int. J. Refract. Met. Hard Mater..

